# The Effects of Supraphysiological Estrogen Levels Observed During In Vitro Fertilization Treatment on Cardiac Electrophysiology

**DOI:** 10.1111/anec.70160

**Published:** 2026-02-20

**Authors:** Burhan Savaş, Dilay Gök Korucu, Gülay Gök

**Affiliations:** ^1^ Department of Obstetrics and Gynecology University of Health Sciences Konya City Hospital Konya Turkey; ^2^ IVF Unit, Department of Obstetrics and Gynecology University of Health Sciences Konya City Hospital Konya Turkey; ^3^ Department of Cardiology Medipol University Istanbul Turkey

**Keywords:** cardiac arrhythmia, controlled ovarian hyperstimulation, electrocardiography, estradiol, fertilization in vitro, ventricular repolarization

## Abstract

Supraphysiological estradiol (E2) levels during in vitro fertilization (IVF) may influence cardiac electrophysiology, but this effect is not well defined. We prospectively evaluated the impact of high E2 concentrations induced by controlled ovarian hyperstimulation (COH) on electrocardiographic (ECG) parameters of atrial conduction and ventricular repolarization. To our knowledge, this is the first study to examine these detailed electrophysiological indices in the context of supraphysiological E2. This prospective observational study included 62 healthy women undergoing an IVF protocol. Standard 12‐lead ECGs and serum hormone levels were obtained before stimulation (baseline E2 < 50 pg/mL) and at peak E2 concentration. Changes in PR interval, corrected QT (QTc) interval, J‐Tpeak (J‐Tp) distance, and Tpeak‐Tend (Tp‐e) interval were analyzed with paired tests. COH produced a marked rise in E2 and significant prolongations in all cardiac intervals. From baseline to peak E2, PR increased from 138.50 ± 20.59 to 144.53 ± 19.31 ms (*p* = 0.007), QTc from 410.0 to 422.0 ms (*p* = 0.003), J‐Tp from 208.74 ± 30.52 to 216.56 ± 30.98 ms (*p* = 0.005), and Tp‐e from 80.00 to 83.50 ms (*p* < 0.001). Supraphysiological E2 levels during IVF are associated with alterations in cardiac electrophysiology, including prolongation of atrial conduction and ventricular repolarization intervals. Although values remained within normal limits in healthy women, the observed QTc and Tp‐e prolongation suggest a potential pro‐arrhythmic substrate and highlight the importance of cardiac monitoring in IVF patients, particularly those with cardiovascular risk.

**Trial Registration:** ClinicalTrials.gov identifier: NCT07007416

## Graphical abstract

Supraphysiological estradiol levels during IVF significantly prolong PR, QTc, J‐Tpeak, and Tp‐e intervals on ECG, suggesting altered atrial and ventricular conduction. These findings highlight a potential pro‐arrhythmic effect of high E2 states and underscore the need for cardiac monitoring in at‐risk IVF patients.

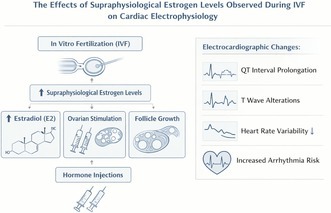

**Abbreviations**
COH: controlled ovarian hyperstimulation
E2: estradiol
ECG: electrocardiography
IVF: in vitro fertilization
J‐T p: J‐Tpeak
QTc: corrected QT
Tp‐Te: Tpeak T end

## 1 Introduction

Cardiovascular diseases remain the predominant cause of global mortality, with a persistently increasing burden. As reported by the World Health Organization, these conditions were responsible for approximately one‐third of worldwide deaths in 2016, with projections indicating an additional 3 million fatalities by 2030 (da Silva et al. 2021).

The influence of gender on the incidence of cardiac arrhythmias has been extensively documented in the literature (Larsen and Kadish 1998). Given that several electrophysiological parameters fluctuate throughout the menstrual cycle, observed variations in ECG measurements and arrhythmia susceptibility are hypothesized to be mediated by sex hormones, particularly E2 and progesterone (PRG) (Tsougos et al. 2021). Although E2 levels in women fluctuate at physiologically low levels during the normal menstrual cycle, the peak levels of E2 are observed during gonadotropin therapy used during IVF treatments. In women, physiological E2 levels typically range from 27 to 123 pg/mL during the follicular phase and 96–436 pg/mL in the luteal phase of the menstrual cycle. However, during COH as part of IVF therapy, E2 concentrations can rise significantly, reaching approximately 4000 pg/mL (Yiginer et al. 2018; Tatchum‐Talom et al. 2002).

Experimental and clinical studies have demonstrated that sex hormones significantly influence cardiac electrophysiology, modulating both action potential characteristics and ECG parameters. Research findings indicate that endogenous testosterone and PRG exert QT interval‐shortening effects, while E2 appears to prolong QT duration (Yiginer et al. 2018; Rodriguez et al. 2001). The QT interval, representing myocardial repolarization duration on ECG, serves as a critical electrophysiological marker, with its prolongation being strongly associated with increased risk of life‐threatening ventricular arrhythmias (Tse et al. 2016). In addition, the J‐Tpeak interval, a key marker of early repolarization, demonstrates significant shortening during peak E2 levels. This observation supports the hypothesis that E2 may exert a protective effect against arrhythmias associated with early repolarization abnormalities, while potentially predisposing to late repolarization disturbances (Salama and Bett 2014).

This study aims to investigate the impact of hormonal fluctuations induced by IVF treatment, particularly supraphysiological E2 concentrations, on cardiac electrophysiology. Through this investigation, we aim to contribute to the literature by elucidating the potential electrophysiological consequences of supraphysiological E2 levels in high‐risk populations, including patients with: preexisting cardiac disease, family history of sudden cardiac death, or concomitant proarrhythmic medication use.

## 2 Materials and Method

This investigation was conducted as a prospective observational study. The study population comprised 62 patients undergoing IVF treatment at Health Sciences University, Konya Hospital IVF Center between December 2024 and January 2025. Electrocardiograms and blood samples were collected and analyzed from the patients both before and after ovarian hyperstimulation treatment. Eligibility required patients undergoing COH for IVF with various indications, normal findings on cardiac examination, biochemical laboratory tests, and myocardial repolarization and depolarization parameters. Patients meeting any of the following criteria were excluded from the study: (1) technically inadequate ECG recordings; (2) obesity, defined as a body mass index (BMI) exceeding 30 kg/m^2^; (3) current use of medications unrelated to infertility treatment; (4) a family history of sudden cardiac death in first‐degree relatives; or (4) a preexisting diagnosis of chronic metabolic or cardiovascular disorders, including diabetes mellitus, hypertension, or other relevant conditions; (5) refusal to provide study consent.

This study received ethical approval from the Institutional Review Board of Konya Karatay University Faculty of Medicine (approval code: 2024/026; date: 26.09.2024). Prior to participation, all enrolled subjects provided written informed consent in compliance with the study protocol. The investigation was conducted in accordance with internationally recognized ethical standards, including adherence to the principles outlined in the Declaration of Helsinki.

### 2.1 Controlled Ovarian Hyperstimulation Protocol

Hormonal baselines and ultrasounds were conducted on the second or third day of the menstrual cycle for all participants, initiating a short flexible antagonist IVF protocol thereafter. Individual doses of rec‐FSH (150–225 IU of Gonal f, Merck, Italy) or a combination of rec‐FSH and hMG (75–150 IU of Merional, IBSA, Switzerland) were administered, with adjustments based on BMI. Follicle monitoring occurred every 2–3 days via transvaginal ultrasound. Once follicles reached 13–14 mm, a flexible dose of the antagonist cetrorelix (Cetrotide, Merck, Italy) was introduced. Follicle growth continued until reaching 17–18 mm, at which point 250 IU of rec‐hCG (Ovitrelle, Merck, Italy) was administered to trigger final maturation. Oocyte retrieval was performed under appropriate anesthesia 36 h later, followed by intracytoplasmic sperm injection (ICSI) using M2 oocytes.

### 2.2 Study Protocol

ECG assessments and venous blood sampling were conducted at two time points for all participants: (1) prior to initiating ovarian hyperstimulation therapy (baseline phase with E2 levels < 50 pg/mL), and (2) during the follicular maturation phase immediately preceding human chorionic gonadotropin (hCG) administration (at the time of peak E2 concentration). Venous blood samples were collected uniformly between 08:30 and 09:30 under standardized conditions. Hemogram parameters and some electrolyte values were compared before and after treatment. To control for circadian variations in cardiac repolarization, 12‐lead electrocardiograms were subsequently acquired at approximately 10:00 on the same morning, immediately following blood sampling. Following a 10‐min supine rest period with standard electrode placement, 12‐lead ECG were acquired using a Schiller AT‐102 system (Schiller AG, Baar, Switzerland) with the following parameters: paper speed of 50 mm/s, amplification of 0.1 mV/mm, and maintained supine positioning throughout recording. All ECG were digitized and transferred to a dedicated workstation to minimize measurement error. Following 400% magnification using Adobe Photoshop CC (v22.0), on‐screen manual measurements were performed by a blinded cardiologist who was independent of the study team.

### 2.3 ECG Measurements

The analyzed ECG parameters included heart rate (HR), PR interval, RR interval, QRS duration, QT interval, QTc, J‐Tp distance, and Tp‐e distance. These parameters were compared between the baseline day of treatment, when serum E2 levels were at their lowest, and the day of the hCG trigger, when E2 levels were at their peak. Quantification of the electrocardiographic ventricular repolarization involving the T‐U wave complex and any abnormal finding (including technical errors) on a 12 lead ECG was performed by one experienced observer. If there were any U wave or ST segment changes in any leads, they were noted separately (Merri et al. 1989).

The QT interval was assessed using the tangent method to define the end of the T wave in all measurable leads, and the longest QT value was recorded in accordance with the AHA/ACCF/HRS 2009 recommendations. Lead DII was preferred for analysis as it most consistently provided optimal T‐wave morphology; when DII was unsuitable, alternative leads (most commonly V5 or V6) were used. Although lead DII was the primary lead used for interval analysis, precordial leads (V5–V6) were evaluated in cases where T‐wave morphology in DII was indistinct. This approach ensured optimal visualization and reproducibility of Tp–Te and J–Tp intervals. All ECG parameters were manually measured twice by the same blinded cardiologist at an interval of at least 2 weeks, and intraobserver variability was below 5% for QT, Tp–Te, and J–Tp intervals, confirming high reproducibility of measurements.

#### 2.3.1 PR Interval

The interval from the beginning of the P wave to the beginning of the QRS complex.

#### 2.3.2 J‐Tp

The positive deviation at the junction of the QRS complex, which reflects ventricular activity, and the ST segment that connects this complex and the T wave is called the J point. The J‐Tp distance refers to the time from the J point to the peak of the T wave and reflects the early repolarization phase.

#### 2.3.3 QT Interval

The part from the beginning of the QRS complex to the end of the T wave was determined as the QT interval.

#### 2.3.4 QTc

Bazett's formula (QTc = QT/√RR) was used to calculate the QTc interval (Sagie et al. 1992).

#### 2.3.5 Tp‐Te

It is the distance from the peak to the end of the T wave. It represents transmural repolarization (Xia et al. 2005).

The measurements were done mostly from the DII derivations. Cases where the clear waves could not be determined or if there were any technical errors were excluded from the study.

### 2.4 Statistical Analysis

IBM SPSS Statistics version 27 was used for data analysis. Continuous variables were expressed as mean ± standard deviation when normally distributed and as median (25th–75th percentile) when non‐normally distributed, whereas categorical variables were presented as frequencies and percentages. For comparisons of blood parameters and electrocardiographic variables before and after treatment, the paired‐samples *t*‐test was applied for normally distributed data, and the Wilcoxon signed‐rank test for non‐normally distributed data. Effect sizes were calculated for both the paired‐samples *t*‐test and the Wilcoxon signed‐rank test. Correlations between continuous variables were assessed using Pearson's product–moment correlation when both variables were normally distributed, and Spearman's rank correlation when at least one variable was not normally distributed. A *p*‐value < 0.05 was considered statistically significant for all analyses.

## 3 Results

Table 1 shows the descriptive information and distribution of blood parameters of all the participants in the study. BUN (blood urea nitrogen), creatinine, TSH (thyroid stimulating hormone), E2, WBC (white blood count), Hb (hemoglobin), Hct (hematocrit), Na (sodium), and LH (luteinizing hormone) values were found to be significantly different before and after gonadotropin treatment (*p* < 0.001, *p* < 0.01, *p* < 0.05). BUN, creatinine, HB, Hct, Na, and LH values decreased from before to after treatment, while TSH, E2, and WBC values increased. Several hematological and biochemical parameters, including BUN, creatinine, Hb, Hct, and Na, demonstrated a statistically significant reduction following treatment, most likely attributable to estrogen‐induced fluid retention and consequent hemodilution. A comparison of blood parameters before and after treatment of participants receiving gonadotropin therapy is described in Table 2.

**TABLE 1:** Distribution of descriptive characteristics and blood parameters of participants receiving gonadotropin therapy (*N* = 62).

Descriptive characteristics			*N*	%
	Unexplained Infertility		18	29.0
Indication for IVF	Male factor		21	33.9
	Poor ovary reserve		23	37.1
	**Min**.	**Max**.	**Mean**	**SD**
Age (years)	21	40	31.06	5.37
BMI (kg/m^2^)	22	29	26.85	1.87
**Blood parameters**	**Min**.	**Max**.	**Mean**	**SD**
WBC (10^3^/μL)	3.87	16.68	7.50	2.37
Hemoglobin (g/dL)	9.6	15.2	13.01	1.66
Hematocrit (%)	28.6	46.4	41.15	7.44
AST (U/L)	7	38	18.66	5.37
ALT (U/L)	8	64	17.21	9.46
BUN (mg/dL)	4	15	9.37	2.36
Uric acid (mg/dL)	1.9	18.0	4.44	2.02
Creatinine (mg/dL)	0.40	0.86	0.63	0.09
Sodium (Na, mmol/L)	135	144	140.03	1.98
Potassium (K, mmol/L)	3.6	9.0	4.55	0.68
Prolactin (μg/L)	7.8	23.2	15.93	4.46
TSH (mU/L)	0.40	2.57	2.17	0.98
FSH (U/L)	3.33	17.60	7.34	2.66
LH (U/L)	1.96	19.40	7.17	3.30
Estradiol (ng/L)	22	50	39.01	7.92

Abbreviations: ALT, alanine aminotransferase; AST, aspartate transferase; BMI, body mass index; BUN, blood urea nitrogen; dL, deciliter; FSH, follicle stimulating hormone; L, liter; LH, luteinizing hormone; mg, milligram; mmol, millimole; ng, nanogram; TSH, thyroid stimulating hormone; U/L, units per liter; WBC, white blood count.

**TABLE 2:** Pretest and posttest analysis of blood parameters before and after treatment in participants receiving gonadotropin therapy (*N* = 62).

Blood parameters	Before treatment		After treatment		*t*	*p* ^a^	Effect size
	$\bar{x}$ ± SD		$\bar{x}$ ± SD				
BUN (mg/dL)	9.37 ± 2.36		8.51 ± 2.76		2.256	**0.028**	0.287
Creatinine (mg/dL)	0.63 ± 0.09		0.60 ± 0.09		3.090	**0.003**	0.392
Prolactin (μg/L)	15.93 ± 4.46		15.69 ± 6.60		0.263	0.793	0.033
TSH (mU/L)	2.17 ± 0.98		2.50 ± 1.06		−2.894	**0.005**	0.368
Estradiol (ng/L)	39.01 ± 7.92		2619.97 ± 1630.62		−12.453	**< 0.001**	1.582
	**Med**. **(25th–75th p.)**	$\bar{x}$ **± SD**	**Med**. **(25th–75th p.)**	$\bar{x}$ **± SD**	***Z***	***p*** ^b^	**Effect size**
WBC (10^3^/μL)	6.98 (5.91–8.76)	7.50 ± 2.37	9.44 (8.41–10.87)	9.60 ± 2.16	−5.711	**< 0.001**	0.725
Hemoglobin (g/dL)	13.50 (12.48–14.20)	13.01 ± 1.66	12.95 (11.75–13.50)	12.50 ± 1.51	−5.058	**< 0.001**	0.653
Hematocrit (%)	41.20 (39.10–43.53)	41.15 ± 7.44	39.65 (36.65–42.10)	43.20 ± 32.19	−4.468	**< 0.001**	0.572
AST (U/L)	18.00 (15.00–22.00)	18.66 ± 5.37	18.00 (14.00–23.00)	20.65 ± 13.58	−0.197	0.844	0.027
ALT (U/L)	15.00 (12.00–19.00)	17.21 ± 9.46	15.50 (12.00–21.50)	21.36 ± 16.92	−1.055	0.291	0.137
Uric acid (mg/dL)	4.25 (3.48–5.05)	4.44 ± 2.02	4.15 (3.40–4.70)	4.06 ± 1.01	−1.229	0.219	0.163
Sodium (Na, mmol/L)	140.0 (139.0–141.0)	140.03 ± 1.98	139.0 (137.8–140.0)	136.92 ± 15.47	−3.611	**< 0.001**	0.487
Potassium (K, mmol/L)	4.50 (4.20–4.60)	4.55 ± 0.68	4.40 (4.20–4.60)	4.62 ± 1.64	−1.332	0.183	0.183
FSH (U/L)	6.88 (5.57–8.23)	7.34 ± 2.66	7.10 (5.87–9.05)	8.16 ± 3.67	−1.524	0.128	0.197
LH (U/L)	6.53 (5.10–8.43)	7.17 ± 3.30	3.86 (2.36–6.72)	5.22 ± 4.38	−3.744	**< 0.001**	0.475

Abbreviations: 25–75, interquartile range; Med, median; SD, standard deviation; $\bar{x}$, mean.

^a^ Paired *t*‐test.

^b^ Wilcoxon signed‐rank test.

PR interval, J‐Tp distance, QTc, and Tp‐e values of the participants receiving gonadotropin treatment were found to differ significantly before and after treatment (*p* < 0.007, *p* < 0.005, *p* < 0.003, *p* < 0.001). Although a statistically significant increase in QTc interval was observed after treatment, all QTc values remained below 460 ms, and no participant met the criteria for QTc prolongation. PR, J‐Tp, QTc, and Tp‐e values increased from pretreatment to posttreatment (PR: 138.50 ± 20.59 vs. 144.53 ± 19.31, J‐Tp: 208.74 ± 30.52 vs. 216.56 ± 30.98, QTc: 410.0 [396.0–426.3] vs. 422.0 [405.3–436.0], Tp‐e: 80.00 [70.00–82.00] vs. 83.50 [80.00–89.25]). Table 3 shows the values of ECG parameters before and after gonadotropin treatment.

**TABLE 3:** Pre‐test and post‐test analysis of ECG parameter values before and after treatment in participants receiving gonadotropin therapy (*N* = 62).

ECG parameters	Before treatment		After treatment		*t*	*p* ^a^	Effect size
	$\bar{x}$ ± SD		$\bar{x}$ ± SD				
HR (bpm)	79.24 ± 14.22		81.18 ± 14.45		−1.510	0.136	0.192
P‐R (ms)	138.50 ± 20.59		144.53 ± 19.31		−2.811	**0.007**	0.357
J‐Tp (ms)	208.74 ± 30.52		216.56 ± 30.98		−2.943	**0.005**	0.374
	**Med**. **(25th–75th p.)**	$\bar{x}$ **± SS**	**Med**. **(25th–75th p.)**	$\bar{x}$ **± SS**	***Z***	***p*** ^b^	**Effect size**
R‐R (ms)	76.00 (68.75–85.25)	77.87 ± 13.99	73.00 (67.25–83.00)	76.29 ± 14.56	−1.758	0.079	0.239
QRS (ms)	86.50 (81.00–91.25)	86.92 ± 9.30	87.00 (81.00–92.00)	100.97 ± 103.71	−0.191	0.849	0.025
QT (ms)	357.0 (342.8–378.5)	364.76 ± 37.58	363.0 (347.0–378.3)	363.95 ± 26.18	−0.632	0.527	0.081
QTc (ms)	410.0 (396.0–426.3)	412.97 ± 26.17	422.0 (405.3–436.0)	418.79 ± 21.35	−2.928	**0.003**	0.375
Tp‐Te (ms)	80.00 (70.00–82.00)	77.44 ± 13.66	83.50 (80.00–89.25)	84.26 ± 10.97	−5.291	**< 0.001**	0.683

Abbreviations: 25–75, interquartile range; bpm, beats per minute; HR, heart rate; Med, median; ms, millisecond; SD, standard deviation; $\bar{x}$, mean.

^a^ Paired *t*‐test.

^b^ Wilcoxon signed‐rank test.

## 4 Discussion

The risk of ventricular arrhythmia is doubled in women relative to men. Female sex is additionally established as an independent risk factor for congenital long QT syndrome and drug‐induced torsades de pointes, one of the causes of sudden cardiac death (De Vecchis et al. 2018).

Our study demonstrated that supraphysiologic E2 levels following COH lead to electrophysiological alterations, manifesting as prolonged PR interval, J‐Tp distance, QTc interval, and Tp‐e interval.

The established normal QTc interval range in females is 360–460 ms. A prolonged QTc is defined as > 450 ms in males and > 460 ms in females. A QTc exceeding 500 ms is associated with a twofold to threefold increased risk of Torsades de Pointes and/or other ventricular arrhythmias (Cohagan and Brandis 2025). In our study, the baseline QTc interval measured 422.0 ms (405.3–436.0), within normal physiological range. However, gonadotropin administration induced a statistically significant QTc prolongation compared to pretreatment values. This finding contrasts with Üçkuyu et al.'s (2012) report of unchanged QTc intervals during ovulation induction. We propose this divergence stems from threshold‐dependent E2 effects—while Uçkuyu's cohort demonstrated peak E2 levels of 684.1 ± 219.8 pg/mL, our data show QT prolongation occurring at substantially higher concentrations (2619.97 ± 1630.62 pg/mL). In addition, Yiginer et al. (2018) evaluated the QTc interval in 59 IVF patients. Changes in supraphysiologic E2 levels (1656 ± 878 pg/mL) were statistically significant, but were not clinically significant in terms of arrhythmia risk because the values obtained were within the reference range. These results demonstrate that supraphysiological E2 levels achieved during IVF can prolong QTc intervals, warranting careful cardiac monitoring for potential ventricular arrhythmias in high‐responder patients.

The Tp‐e interval, when prolonged, independently correlates with ventricular arrhythmia and sudden cardiac death, offering diagnostic utility when the QT interval is either within normal limits or unmeasurable due to QRS prolongation (Tse et al. 2017; Lester and Paglialunga 2025). Therefore, the Tp‐e interval acts as a noninvasive indicator of arrhythmogenesis (Castro‐Torres et al. 2015). In our study, we also found a statistically significant increase in the Tp‐e interval at peak E2 level. Even Yiginer et al. (2018) found this value to be increased in supraphysiologic E2 levels, although not statistically significant. To our knowledge, no study has investigated the relationship between Tp‐e interval and E2 levels so far, and our study adds to the literature that high E2 levels may have arrhythmogenic effects.

The J‐Tp interval, the early repolarization parameter in an ECG, detected as prolonged in our study (208.74 ± 30.52 ms vs. 216.56 ± 30.98, *p* = 0.005). However, in contrast to our study, Doğan et al. in (2016) investigated repolarization parameters during the physiological menstrual cycle and found that the mean J‐Tp distance was 201.1 ± 28.6 ms during the menstrual period, while this distance was 193 ± 27.7 ms during periods when E2 levels increased. Compared to the findings of Doğan et al. during a physiological cycle, the higher levels of E2 formed under the influence of exogenous gonadotropins may be the reason for this discrepancy. Calcium channels play a key role in early repolarization as the most significant active ion channels. While some research indicates that E2 inhibits calcium channels, Nakagawa et al. (2006) found that progesterone has a more pronounced effect on the action potential. The findings of Nakagawa et al. suggest that elevated serum progesterone levels correlate with a shortened QT interval during the luteal phase. This reduction in the J‐Tp interval may be attributed to the effects of progesterone. The influence of E2 on action potential remains debated. While some studies have demonstrated that 17‐β estradiol shortens action potential duration in guinea pig ventricular muscle (Grohe et al. 1996; Liu et al. 1997), Berger et al. (1997) reported a prolongation of the action potential with 17‐β estradiol.

The P‐R interval, measured from the beginning of the P wave to the onset of the QRS complex, represents the time it takes for the atrial impulse to travel through the atrioventricular node. This delay allows time for ventricular filling to take place (Goldberger 2018). The P‐R interval has been shown to be prolonged in conditions such as atrial fibrillation, hypertension, diabetes mellitus, and cardiovascular diseases (Yazici et al. 2007). However, the number of studies investigating the influence of hormonal effects on the PR interval in women is limited. The first study in the literature to examine the variation of the P‐R interval with the hormonal cycle was conducted by Yiginer et al. (2018). Their study, which included 59 infertile female participants, investigated the effects of supraphysiological E2 levels on ventricular repolarization. In their study, no correlation was found between the change in E2 levels and the P‐R interval. In contrast, our study demonstrated a statistically significant increase in the P‐R interval from a pretreatment value of 138.5 ± 20.59 ms to 144.53 ± 19.31 ms posttreatment. This discrepancy may be attributable to the higher mean E2 levels in our study cohort.

We acknowledge that the Bazett's correction formula may overestimate the QT interval at elevated heart rates. However, since the mean heart rate values in our cohort were within the normal physiological range, this effect is likely minimal. Moreover, Bazett's correction was selected to maintain methodological consistency with prior studies examining estrogen‐related electrophysiological changes.

The relatively small number of patients is one of our main limitations. Another limitation of our study is that heart rate correction for Tp–Te and J–Tp intervals was not performed. However, since heart rate remained within a narrow physiological range without significant variation between measurements, the potential confounding effect of heart rate is expected to be minimal. The study's design captures the acute electrophysiological effects of supraphysiological E2 at a single time point. A significant limitation is the absence of long‐term follow‐up. We did not assess whether the observed ECG changes (e.g., QTc and PR prolongation) are transient and normalize after the IVF cycle, nor did we monitor for the actual incidence of clinical cardiac events in the months following treatment. In addition, The COH protocol allowed for the use of either rec‐FSH alone or a combination of rec‐FSH and hMG. Although this reflects real‐world clinical practice, the inclusion of hMG (which has LH activity) introduces a minor heterogeneity in the stimulation regimen that could be a potential confounding factor. Although performed by a blinded cardiologist to minimize bias, the manual measurement of ECG intervals on magnified digital images may be subject to greater interobserver and intraobserver variability compared to automated, validated software algorithms. This could potentially influence the precision of the measured intervals. A major strength of this study is its prospective design. On the other hand, this research addresses a highly relevant clinical question concerning the safety of IVF. By studying the effects of supraphysiological hormone levels that cannot be observed in a natural menstrual cycle, this study provides valuable insights that could inform the cardiac monitoring and risk assessment of patients undergoing controlled ovarian hyperstimulation, especially those in high‐risk groups.

In conclusion, this prospective study provides clear evidence that the supraphysiological E2 concentrations induced by IVF treatment lead to significant and complex alterations across the heart's electrical cycle. Our findings show a statistically significant prolongation of key ECG intervals, including the PR interval (affecting atrial conduction), the QTc interval, and the Tp‐e interval (affecting ventricular repolarization). This suggests that high E2 states create a potential pro‐arrhythmic substrate by simultaneously impacting multiple phases of cardiac electrophysiology.

Although the absolute values of these markers remained within normal limits in our healthy study population, the observed changes are noteworthy. They highlight a potential vulnerability and underscore the importance of careful patient evaluation prior to IVF. Enhanced cardiac monitoring may be warranted for patients with underlying cardiovascular conditions, a family history of sudden cardiac death, or those using concomitant medications. Further large‐scale, multicenter studies with long‐term follow‐up are essential to determine the clinical significance of these findings and to establish robust risk‐stratification strategies for this growing patient population.

## Author Contributions

D.G.K. and B.S.: Study design, patient management, and manuscript writing/editing. B.S.: Data analysis, patient management. G.G.: Data analysis, patient management. B.S.: Data collection. G.G.: Contributed to and approved of the final version of the manuscript. All of the authors have read and approved the manuscript.

## Funding

The authors have nothing to report.

## Conflicts of Interest

The authors declare no conflicts of interest.

## Data Availability

The datasets generated and analyzed during the present study are available from the corresponding author on reasonable request.
